# Temporal–Spatial Fluctuations of a Phytoplankton Community and Their Association with Environmental Variables Based on Classification and Regression Tree in a Shallow Temperate Mountain River

**DOI:** 10.3390/microorganisms12081612

**Published:** 2024-08-07

**Authors:** Wang Tian, Zhongyu Wang, Haifei Kong, Yonglan Tian, Tousheng Huang

**Affiliations:** Research Center for Engineering Ecology and Nonlinear Science, North China Electric Power University, Beijing 102206, China; tianwang@ncepu.edu.cn (W.T.); konghaifei@ncepu.edu.cn (H.K.); yonglantian@ncepu.edu.cn (Y.T.); tous_huang@ncepu.edu.cn (T.H.)

**Keywords:** phytoplankton, species diversity, abundance, environmental variables, classification and regression tree

## Abstract

The effects of environmental factors on phytoplankton are not simply positive or negative but complex and dependent on the combination of their concentrations in a fluctuating environment. Traditional statistical methods may miss some of the complex interactions between the environment and phytoplankton. In this study, the temporal–spatial fluctuations of phytoplankton diversity and abundance were investigated in a shallow temperate mountain river. The machine learning method classification and regression tree (CART) was used to explore the effects of environmental variables on the phytoplankton community. The results showed that both phytoplankton species diversity and abundance varied fiercely due to environmental fluctuation. *Microcystis aeruginosa*, *Amphiprora* sp., *Anabaena oscillarioides*, and *Gymnodinium* sp. were the dominant species. The CART analysis indicated that dissolved oxygen, oxidation-reduction potential, total nitrogen (TN), total phosphorus (TP), and water temperature (WT) explained 36.00%, 13.81%, 11.35%, 9.96%, and 8.80%, respectively, of phytoplankton diversity variance. Phytoplankton abundance was mainly affected by TN, WT, and TP, with variance explanations of 39.40%, 15.70%, and 14.09%, respectively. Most environmental factors had a complex influence on phytoplankton diversity and abundance: their effects were positive under some conditions but negative under other combinations. The results and methodology in this study are important in quantitatively understanding and exploring aquatic ecosystems.

## 1. Introduction

Phytoplankton are the most important primary producers, and they play a key role in maintaining the productivity and stability of aquatic ecosystems [1,2,3]. Due to their small body size, phytoplankton generally have a high growth rate, short generation time, fast trophic transfer ability, and environmental sensitivity [4,5,6,7]. Eutrophication and environmental degradation caused by global climate change and human activities have a catastrophic influence on phytoplankton communities, such as algae blooms and a large number of species extinctions [7,8,9,10]. Therefore, analyzing the determining variables of phytoplankton and predicting their variations in a fluctuating environment are important in maintaining a healthy aquatic ecosystem.

The prediction of phytoplankton is difficult due to their special ecophysiological characteristics and the complexities of the environment [6,7]. In natural aquatic ecosystems, dozens of environmental variables affect the growth of phytoplankton, including water temperature (WT), light, dissolved oxygen (DO), pH, turbidity (Tur), water transparency, electrical conductivity (EC), various forms of nitrogen and phosphorus, and so on [11]. Essential nutrients and temperature can promote phytoplankton abundance to a saturation level [12,13]. Phytoplankton remove dissolved inorganic carbon and produce oxygen through photosynthesis, which leads to higher pH and DO concentrations [14,15,16]. Therefore, DO concentration is usually used as an indicator to estimate primary productivity and aquatic ecosystem respiration rates [14,15]. However, an excess of phytoplankton or algae blooms would result in low DO concentrations and exclude the survival of other taxa [9,10,17]. The effects of environmental factors on phytoplankton are not simple linear relationships. Hinga [18] and Hansen [19] found phytoplankton growth rate had a unimodal relationship with water pH and reached the maximum value when pH ranged from 8.3 to 8.5. Phytoplankton diversity also has complex responses to environmental changes, e.g., the species number of phytoplankton generally increases with nutrient concentrations in oligotrophic systems, while it decreases in eutrophic environments due to the “paradox of enrichment” [20,21,22]. Environmental variables also have interactive effects on the growth of phytoplankton, such as the ratio between nitrogen and phosphorus [13,23,24]. The environmental condition of phytoplankton in a local natural ecosystem is the combination of these factors which fluctuate all the time [7,11,13].

Like most complex systems, the prediction of phytoplankton in natural aquatic ecosystems is difficult. Statistical approaches such as linear regression, polynomial regression, and nonlinear analysis can help us understand the quantitative relationship between environmental variables and phytoplankton to some extent. Restricted sorting methods developed on the basis of correspondence analysis, e.g., redundancy analysis and canonical correspondence analysis, are powerful methods to analyze the effects of environmental variables on biological communities [25,26]. Traditional hydrodynamic models were also used to predict the migration and transformation of nutrient elements and the dynamics of phytoplankton [27,28]. Huang et al. [29] used a hydrodynamic phytoplankton model simulating the short term spatial and temporal distribution of phytoplankton in Lake Taihu. Pathak et al. [30] developed a river model based on the Quality Evaluation and Simulation Tool for River Systems to simulate hourly scale phytoplankton growth and its environmental controls. However, restricted sorting and hydrodynamic models are also limited in terms of interpretability due to their black-box nature. Moreover, the influence of environmental factors on phytoplankton may be not simply positive, negative, unimodal, or reversely unimodal. One environmental factor may have a positive influence on phytoplankton under specific conditions, while the effects may be negative or complex in other combinations of environments. Assuming that each factor has two different levels (low and high temperature, nitrogen, phosphorus, etc.), there are more than 2^10^ combinations of all conditions when 10 environmental variables mainly affect phytoplankton. The growth of phytoplankton in each of these combined conditions may be different. However, traditional statistical analysis methods have difficulty identifying the complex and combined effects of environmental variables on phytoplankton.

In recent years, many new statistical methods based on machine learning techniques have been used to explore the influencing factors of phytoplankton and predict their variations. Bourel et al. [31] used random forests, boosting, and support vector machines and their combinations to predict the presence–absence of marine phytoplankton. Jeong et al. [32] predicted the time series of phytoplankton proliferations in a regulated river system based on an artificial neural network. Zhang et al. [33] applied a deep learning approach to analyze the relationship between environmental variables and phytoplankton and predict algae blooms in the coastal waters of East China. Volf et al. [34] also developed descriptive and prediction models of phytoplankton in the northern Adriatic based on machine learning techniques and successfully made a 15-day forecast of phytoplankton concentration. Among the various machine learning methods, classification and regression tree (CART) is a nonparametric technique which is suitable for complex ecological data with a hierarchical structure [35]. CART can statistically demonstrate which factors are particularly important in a model or relationship in terms of explanatory power and variance. Furthermore, regression trees are flexible analytical methods that are robust to non-normally distributed data, nonlinear relationships, and high-order interactions [36]. Meanwhile, it may classify phytoplankton communities based on their characteristics caused by different combinations of environmental factors. Thus, CART may be very suitable to analyze the driving variables of phytoplankton and predict their variations on a local scale.

In the present study, the main environmental factors and phytoplankton community were investigated bimonthly over two years in River Taizicheng, a shallow temperate mountain river. The temporal and spatial patterns of environmental factors and phytoplankton community diversity and abundance were analyzed. CART was used to explore the main driving environmental factors of phytoplankton, calculate the contribution of each factor, and test whether it could be used as a prediction method for phytoplankton. The purpose of the present research is to (1) reveal the temporal–spatial distributions of a phytoplankton community in a fluctuating environment, (2) explore their driving environmental variables, and (3) identify the complex and combined effects of environmental variables on phytoplankton by a machine learning method.

## 2. Materials and Methods

### 2.1. Study Area

Taizicheng is a small mountain river located in Chongli District, Zhangjiakou City, Hebei Province, China. The length of the river is about 27.0 km, and the average width is 2.0 m. The depth of the river is lower than 0.5 m in most parts. The elevation of the Taizicheng River basin varies between 1329 and 2172 m above sea level. The river basin is covered by mountains, and the vegetation coverage reaches more than 65%. *Betula platyphylla Suk* and *Larix thibetica* forests are the main forest types. The river has a climate of temperate monsoon, and annual average temperature varies between −0.3 °C and 6.7 °C. The average temperature of the river basin is about 18.4 °C in summer and −12.0 °C in winter. The freezing and snowfall period in the region lasts from November to March. The boundary of the river catchment is formed by the ridgelines of surrounding mountains, and the area of the catchment is about 235 km^2^. On the whole, there is little human activity in the area, with only a few small villages at the upstream and midstream of the river. The river was rarely studied until the area became a core site of the 2022 Winter Olympics. During the research, a road was built along the river, which may influence the water quality of the river. Underground spring, ice- and snow-melting water, and mountain runoff after precipitation are the main water sources of the river. The river flows into River Qingshui and eventually into River Yanghe. In the river, there are nearly no fish species, and plankton dominates the biological community.

### 2.2. Sampling and Measurements

**Figure 1:**
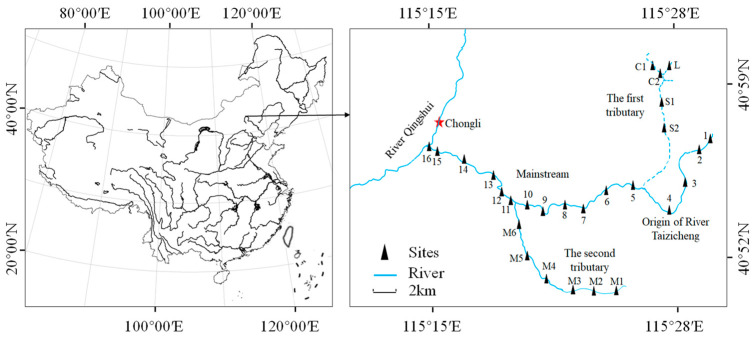
Location of the mountain river and the sampling sites. Labels 1−16, M1−M6, C1, C2, S1, S2, and L are the numbers of sampling sites.

A total of 27 sampling sites were evenly set in the river, as shown in Figure 1. The main water environmental factors and phytoplankton were investigated in April, June, August, and October 2019 and 2020. In December and February, some parts of the river were covered by ice, and sampling was paused.

Environmental variables of WT (°C), pH, DO (mg/L), EC (uS/cm), and oxidation-reduction potential (ORP, mV) were measured using YSI Professional Plus (YSI Incorporated, Yellow Springs, OH, USA). The Tur (NTU) at each site was measured using a portable turbidimeter. Water samples (1 L) were collected at each site using a Tygon tube water sampler and kept in acid-cleaned glass bottles at 4 °C before analysis. Potassium persulfate oxidation-UV spectrophotometry and Mo-Sb anti-spectrophotometry were conducted to calculate the density of total nitrogen (TN, mg/L) and total phosphorus (TP, mg/L), respectively [37]. At each site, 3 L phytoplankton samples were taken from the center and surface of the river and divided into 3 samples at each site. Then, each sample was preserved with acidified Lugol’s solution for 48 h and condensed to 30 mL. Phytoplankton samples were identified to species level under a microscope (Type: Axio Imager M2, Carl Zeiss AG, Oberkochen, Germany), and their abundance was calculated [38,39]. In addition, phytoplankton abundance at each site was the average value of the 3 samples.

### 2.3. Statistical Analysis

The significant differences of environmental variables at different months were calculated using a one-way analysis of variance (ANOVA). Kolmogorov–Smirnov and Bartlett’s tests were calculated to examine whether the data were normally distributed and assess the homoscedasticity. Post hoc tests were calculated using Tukey’s honestly significant difference (HSD) at a significance level of 0.05.

The influence of the main environmental factors on phytoplankton species richness and abundance was analyzed using CART. The values or concentrations of WT, DO, EC, pH, Tur, ORP, TN, and TP were entered as explanatory variables. The response variable (phytoplankton species richness or abundance) was continuous. We thus used a regression tree to calculate the effects of environmental factors on phytoplankton or predict their values. The regression tree splits the data based on the calculated regression error (sample variance), i.e., a split that minimizes data variance. Before calculation, the sample variance of all data was calculated through the following equation:

(1) $$E(D)=\frac{1}{N}\sum_{i=1}^{N}(y_{i}-\frac{1}{N}\sum_{j=1}^{N}y_{j}{)}^{2}$$

Here, *N* is the total number of samples used in the calculation; *y_i_* is the value of the phytoplankton species richness or abundance of the *i*th sample. For each of the splits, we chose the environmental variable and its split point that decreased the sample variance to the maximum value based on the following equation:

(2) $$\begin{array}{l} E=E(D)-\frac{N_{1}}{N}E(D_{1})-\frac{N_{2}}{N}E(D_{2})\\ =\frac{1}{N}\sum_{i=1}^{N}(y_{i}-\frac{1}{N}\sum_{j=1}^{N}y_{j}{)}^{2}-\frac{N_{1}}{N}\left[\frac{1}{N_{1}}\sum_{i=1}^{N_{1}}(y_{1i}-\frac{1}{n}\sum_{j=1}^{N_{1}}y_{1j}{)}^{2}\right]-\frac{N_{2}}{N}\left[\frac{1}{N_{2}}\sum_{i=1}^{N_{2}}(y_{2i}-\frac{1}{n}\sum_{j=1}^{N_{2}}y_{2j}{)}^{2}\right]\\ =\frac{1}{N}\sum_{i=1}^{N}(y_{i}-\frac{1}{N}\sum_{j=1}^{N}y_{j}{)}^{2}-\frac{1}{N}\sum_{i=1}^{N_{1}}(y_{1i}-\frac{1}{n}\sum_{j=1}^{N_{1}}y_{1j}{)}^{2}-\frac{1}{N}\sum_{i=1}^{N_{2}}(y_{2i}-\frac{1}{n}\sum_{j=1}^{N_{2}}y_{2j}{)}^{2} \end{array}$$

Here, *D*_1_ and *D*_2_ are the symbols of the two child nodes, while *N_1_* and *N_2_* are the corresponding sample sizes. In order to prevent the existence of an overfitting phenomenon, the regression tree was pruned using the cost-complexity prune method. This method relies on a complexity parameter, *α*, which was calculated based on the following equation:

(3) $$\alpha =\frac{E(n)-E(n_{t})}{\left|n_{t}\right|-1}$$

Here, *E*(*n*) is the error rate of note *n*, and *E*(*n_t_*) is the error rate of the sub-tree rooted at node *n*. $\left|n_{t}\right|$ is the number of leaf nodes of the sub-tree. *E*(*n*) is calculated through Equation (1). During the pruning process, *α* is gradually increased. The pruning process began at the last level (i.e., the terminal nodes), and the child nodes were pruned away if the resulting change in the predicted misclassification cost was less than α times the change in tree complexity. The proportional contribution of each variable on phytoplankton was calculated by the following equation:

(4) $$PC_{i}=q_{i}/\sum_{j=1}^{k}q_{j}$$

Here, *PC_i_* is the proportional contribution of environmental variable *i* on phytoplankton diversity or abundance, *q_i_* is the summed decreased variance when environmental variable *i* is the split point, and *k* is the number of environmental variables in the CART. Phytoplankton abundance and species richness were transformed by ln(x), and the CARTs were calculated through Matlab (version: 9.0.0.341360, R2016a).

## 3. Results

### 3.1. Main Environmental Variables in the River

During the research, water environmental factors showed significant fluctuations with time (Table 1). Average WT varied from 5.19 ± 2.28 °C (mean ± standard deviation) in October 2019 to 15.40 ± 2.66 °C in August 2020. There were significant differences of WT at different measurements (one way ANOVA: *F*_(7,151)_ = 30.44, *p* < 0.001). DO changed fiercely among different months (*F*_(7,151)_ = 116.02, *p* < 0.001), with the average concentrations in April and June 2020 significantly lower than those in other months (all *p* < 0.001, post hoc Tukey’s HSD). The possible reasons for the lower DO concentrations in April and June 2020 were that the main water source of the river was the underground spring and the construction along the river. The river was in a weak alkaline state as indicated by the pH values (Table 1). The average values of both pH and EC kept relatively constant during the study (e.g., *F*_(7,151)_ = 1.11, *p* = 0.361 for EC), while those of water Tur ranged between 102.4 ± 93.8 in June 2019 and 370.4 ± 266.2 in August 2020. The values of ORP continued to decrease with time in the river (*F*_(7,151)_ = 46.60, *p* < 0.001) and reached the minimum value in August 2020. On the whole, the river had high nutrient concentrations, and their values fluctuated temporally. The average concentrations of TN in June and August 2019 were significantly higher than those in other months (all *p* < 0.001, post hoc Tukey’s HSD). The concentrations of TP in spring (April 2019 and 2020) were much higher than other seasons (all *p* < 0.001, post hoc Tukey’s HSD), as shown in Table 1. The relatively high concentrations of nutrients in spring were mainly caused by the melting of snow and ice in the mountains.

### 3.2. Temporal–Spatial Variations of Phytoplankton Species Diversity and Abundance

**Figure 2:**
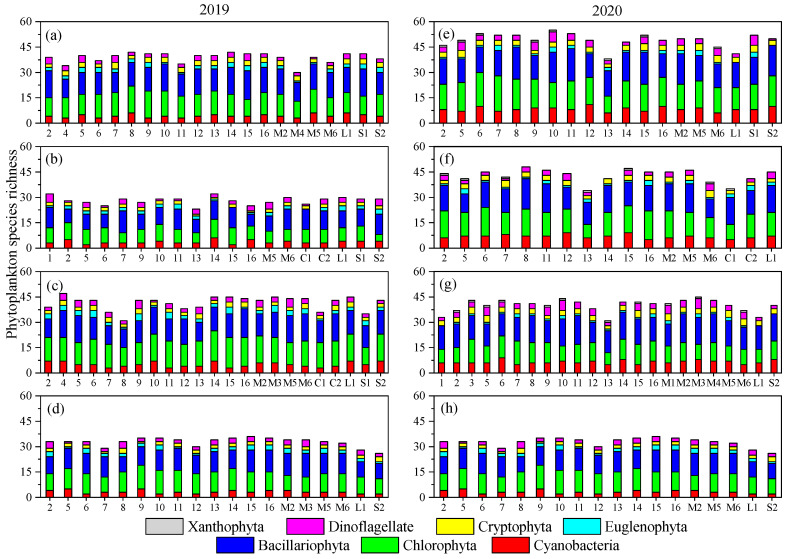
Stacked columns showing the variations in phytoplankton species richness in (**a**) April, (**b**) June, (**c**) August, and (**d**) October 2019 and (**e**) April, (**f**) June, (**g**) August, and (**h**) October 2020.

During the research, 120 phytoplankton species belonging to 64 genera and 7 phyla were identified in the river. The phytoplankton community included 44 Bacillariophyta species, 38 Chlorophyta species, 19 Cyanobacteria species, 10 Euglenophyta species, and so on. Average phytoplankton species richness reached the minimum value (28.11 ± 2.31) in June 2019 and the maximum value (49.11 ± 4.20) in April 2020 (Figure 2). During each of the two years, phytoplankton species richness reached the maximum value in spring (April). The number of Bacillariophyta and Chlorophyta species varied markedly, both temporally and spatially (Figure 2).

**Figure 3:**
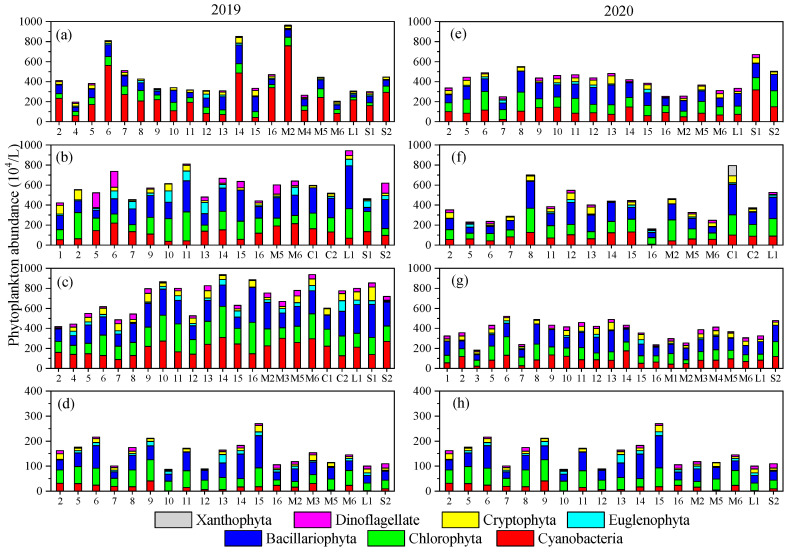
Stacked columns showing the variations in phytoplankton abundance in (**a**) April, (**b**) June, (**c**) August, and (**d**) October 2019 and (**e**) April, (**f**) June, (**g**) August, and (**h**) October 2020.

The average abundance of phytoplankton in the river during the research was 4.11 × 10^6^/L. Phytoplankton abundance was dominated by Bacillariophyta, Cyanobacteria, and Chlorophyta, and their average proportions were 30.64%, 27.08%, and 25.15%, respectively. In April 2019, phytoplankton abundance varied from 1.97 × 10^6^/L to 9.63 × 10^6^/L among different sites. The average abundance proportion of Cyanobacteria reached the maximum value (55.04%) across the two years. *Microcystis aeruginosa* was the dominant species. In June 2019, the average phytoplankton abundance increased to 5.94 × 10^6^/L. The average abundance proportion of Cyanobacteria decreased to 20.22%, while that of Bacillariophyta and Chlorophyta increased to 32.93% and 25.94%, respectively. *Microcystis aeruginosa* and *Amphiprora* sp. became the dominant species in the river. Phytoplankton average abundance reached the maximum value in August 2019 (7.05 × 10^6^/L), and its value at the origin of the river was much lower than those at other sites (Figure 3). The average abundance proportion of Cyanobacteria increased to 28.30% but that of Bacillariophyta decreased to 29.20%. *Microcystis aeruginosa* and *Gymnodinium* sp. were the most abundant phytoplankton species. Average phytoplankton abundance decreased to 1.50 × 10^6^/L in October 2019. Bacillariophyta, Chlorophyta, and Cyanobacteria were also the dominant phyla with average abundance proportions of 36.22%, 32.34%, and 11.80%, respectively. The proportion of Bacillariophyta reached the highest value while that of Cyanobacteria reached the minimum value when the temperature was low. *Anabaena oscillarioides* and *Gymnodinium* sp. became the dominant species. Phytoplankton abundance was much lower in 2020 compared to the same periods in 2019. In April 2020, the average proportion of Cyanobacteria was 25.61%, which was much lower than April 2019. Average phytoplankton abundance continued to decrease from April to October 2020, and the variation amplitude was much smaller than 2019. Bacillariophyta abundance proportions in the four months of 2020 were 31.53%, 35.16%, 33.67%, and 36.43%, respectively, which were significantly higher than those in 2019. *Microcystis aeruginosa* and *Anabaena oscillarioides* were the dominant species in April, June, and August 2020. Furthermore, *Ankistrodesmus falcatus* and *Euglena viridis* were the representative species of Chlorophyta and Euglenophyta during the research.

**Figure 4:**
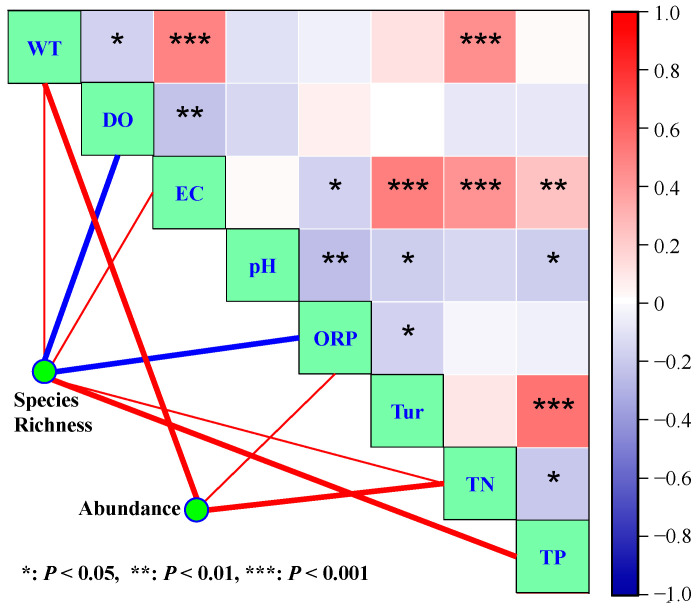
Spearman’s correlation coefficients among the environmental variables and phytoplankton species richness and abundance. Bold red lines: positive correlation, *p* < 0.001; Thin red lines: positive correlation, *p* < 0.05; Bold blue lines: negative correlation, *p* < 0.001.

The correlation analysis results are shown in Figure 4. It can be seen that WT was significantly positively correlated with TN (*r* = 0.46, *p* < 0.001) and EC (*r* = 0.49, *p* < 0.001) but had a negative relationship with DO (*r* = −0.16, *p* < 0.05). EC had strong positive relationships with Tur (*r* = 0.51, *p* < 0.001), TN (*r* = 0.43, *p* < 0.001), and TP (*r* = 0.24, *p* < 0.01). Phytoplankton species richness was significantly positively correlated with TP (*r* = 0.28, *p* < 0.001), EC (*r* = 0.18, *p* < 0.05), WT (*r* = 0.17, *p* < 0.05), and TN (*r* = 0.16, *p* < 0.05) but had strong negative relationships with DO (*r* = −0.55, *p* < 0.001) and ORP (*r* = −0.29, *p* < 0.001). TN (*r* = 0.59, *p* < 0.001), WT (*r* = 0.42, *p* < 0.001), and ORP (*r* = 0.16, *p* < 0.05) had strong positive relationships with phytoplankton abundance. Furthermore, there was also a positive relationship between phytoplankton species richness and abundance (*r* = 0.25, *p* < 0.01).

### 3.3. Effects of Environmental Variables on Phytoplankton Species Diversity

**Figure 5:**
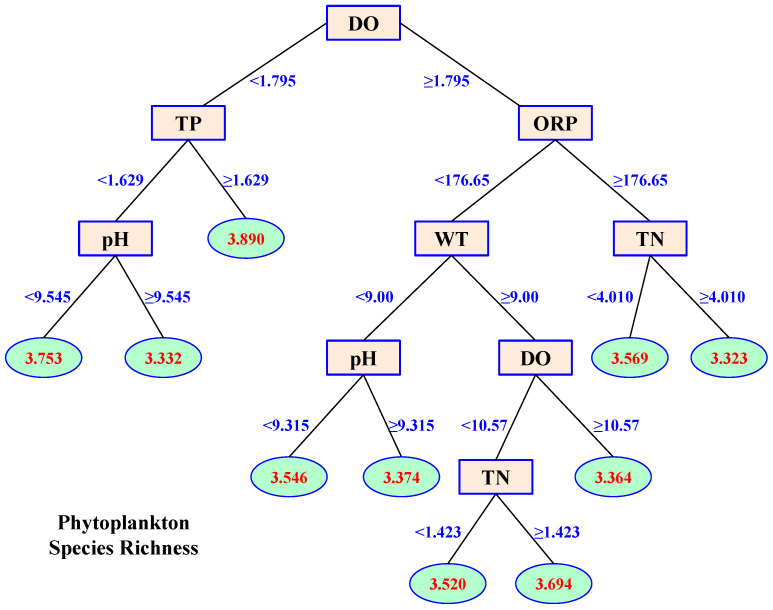
Classification and regression tree (CART) plot of the effects of environmental variables on phytoplankton species richness. The oval frames represent leaf nodes showing the average diversity (ln-transform), and the rectangular boxes show the non-leaf nodes.

The effects of environmental variables on phytoplankton species richness based on CART are shown in Figure 5. The best split point at the first time was DO concentration at 1.795 mg/L. The sample variance (data of phytoplankton were ln-transformed) declined from 0.0349 to 0.0249. Then, the samples were divided into two child nodes (Figure 5). For the node with the lower DO concentration, the best split point was TP concentration at 1.629 mg/L. For the node with the higher DO concentration, the best split point was ORP at a value of 176.65. Using the same method, a regression tree with 32 leaf nodes and 31 non-leaf nodes was obtained through recursive partitioning. The regression tree was pruned based on the cost-complexity prune method to prevent the existence of an over-fitting phenomenon (see Materials and Methods). As a result, the final regression tree reflecting the effects of environmental variables on phytoplankton species richness contained 10 leaf nodes and 9 non-leaf nodes (Figure 5).

In the regression tree, samples with low values of DO (<1.795 mg/L) had relatively high phytoplankton diversity. DO concentration was highly related to water flow. A faster flow in the river was associated with high concentrations of DO but low phytoplankton diversity since they did not have a stable habitat for growth in the mountain river. When the concentration of DO was low, a relatively high TP concentration benefited the species richness of phytoplankton, especially Chlorophyta species. At low values of both DO and TP, a weak alkaline state benefited phytoplankton diversity best (Figure 5). When the concentration of DO was high (≥1.795 mg/L), the lowest phytoplankton diversity appeared at high ORP and TN concentrations, while the highest diversity appeared at low ORP and high WT (Figure 5). Furthermore, the pH splits in the regression tree indicated that phytoplankton diversity was relatively high at low levels of pH. The first TN split was 1.423 mg/L, and it had a positive influence on phytoplankton diversity, while the effect of the second TN split (4.010 mg/L) was negative (Figure 5).

**Figure 6:**
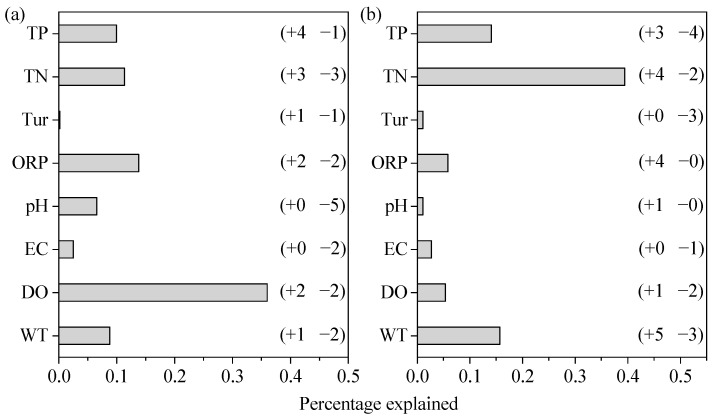
CART analyses identifying the main effects on (**a**) phytoplankton species richness and (**b**) abundance. Bars indicate the proportional contribution of independent variables to the overall explained variance. The signs of the effects and the number of splits that each independent variable contributed are also shown.

The contribution of environmental variables to phytoplankton species richness is shown in Figure 6a. All the variables explained 89.13% of total phytoplankton diversity variance. DO, ORP, TN, TP, and WT were the main environmental variables that influenced phytoplankton diversity in the river, since they explained 36.00%, 13.81%, 11.35%, 9.96%, and 8.80%, respectively, of the variance of phytoplankton species richness (Figure 6). The signs of the effects of TN (+3, −3, i.e., 3 positive splits and 3 negative splits, Figure 6), DO (+2, −2), ORP (+2, −2), TP (+4, −1), WT (+1, −2), and Tur (+1, −1) indicated that the influence of these variables on phytoplankton diversity was complex: their effects were positive under some conditions but negative under other combinations of concentrations. Therefore, some traditional statistical methods may not be suitable for studying environmental effects on phytoplankton diversity, especially in fluctuating environments.

### 3.4. Effects of Environmental Variables on Phytoplankton Abundance

**Figure 7:**
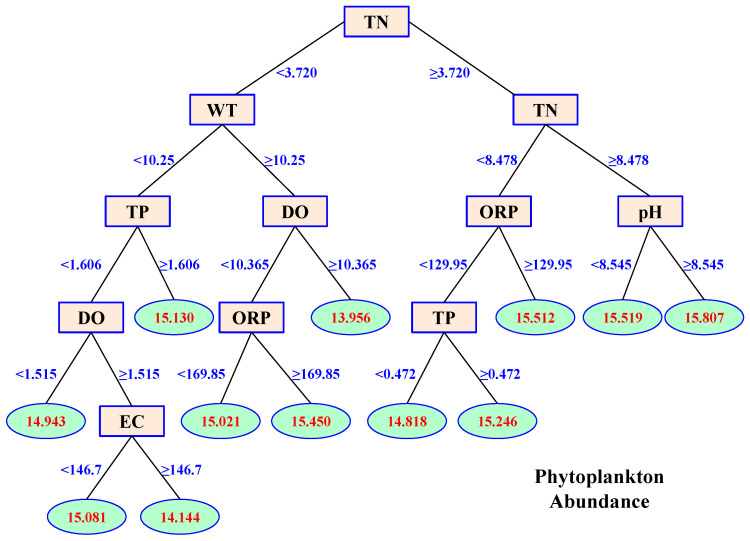
CART plot of the effects of environmental variables on phytoplankton abundance. The oval frames represent leaf nodes showing the average abundance (ln-transform), and the rectangular boxes show the non-leaf nodes.

The effects of environmental variables on phytoplankton abundance based on CART are shown in Figure 7. The results showed the best split point at the first time was TN concentration at 3.720 mg/L. Sample variance (data of phytoplankton abundance were ln-transformed) declined from 0.384 to 0.268. Then, the samples were divided into two child nodes (Figure 7). For the node that had a lower TN concentration, the best split point was WT at 10.25 °C. For the node with the higher TN concentration, the best split point was also TN concentration at 8.478 mg/L. The regression tree obtained through recursive partitioning had 34 leaf nodes and 33 non-leaf nodes. Then, the regression tree was pruned based on the cost-complexity prune method. The final regression tree reflecting the effects of environmental variables on phytoplankton abundance contained 12 leaf nodes and 11 non-leaf nodes (Figure 7).

In the regression tree, TN had a positive influence on the abundance of phytoplankton (Figure 7). When the concentration of TN was low, a relatively high WT benefited phytoplankton abundance most. However, at low TN and high WT, a high concentration of DO inhibited the growth of phytoplankton (Figure 7). When the concentration of TN was high, the largest phytoplankton abundance appeared at high pH values, while the lowest abundance appeared at low ORP and TP (Figure 7).

The contribution of environmental variables on phytoplankton abundance is shown in Figure 6b. All the variables explained 85.14% of total phytoplankton abundance variance. TN, WT, and TP were the main environmental variables that influenced phytoplankton abundance in the river, since they explained 39.40%, 15.70%, and 14.09%, respectively, of phytoplankton abundance variance (Figure 6b). Thus, nutrients and WT were the most important predictors of phytoplankton abundance (Figure 6b). The signs of the effects of WT (+5, −3, i.e., 5 positive splits and 3 negative splits, Figure 6), TP (+3, −4), TN (+4, −2), and DO (+1, −2) indicated that the influence of these variables on phytoplankton abundance was complex: their effects were positive under some conditions but negative under other combinations of concentrations. Therefore, some traditional statistical methods may not be suitable for studying environmental effects on phytoplankton abundance, especially in fluctuating environments.

### 3.5. Model Validation

To validate the precision of the CART model, we used 2/3 of the samples as training data and the remaining 1/3 as test data. The training data and test data were randomly chosen during each of the calculations. We compared phytoplankton species richness (abundance) measured in the test data with that predicted in the CART model. We ran the computation 200 times for the phytoplankton species richness and abundance models separately and calculated their mean errors. The precision of the CART was tested using the following equation:

(5) $$ER=\frac{1}{200}\sum_{i=1}^{200}(\frac{1}{n}\sum_{j=1}^{n}\frac{\left|y_{j}-y_{pred}\right|}{\left|y_{j}\right|})$$

Here, *ER* is the average predicted error of the CART model, *n* is the number of test data, *y_j_* is the measured value of the phytoplankton species richness or abundance of the *j*th sample, and *y_pred_* is the value of phytoplankton species richness or abundance predicted by the CART model.

**Figure 8:**
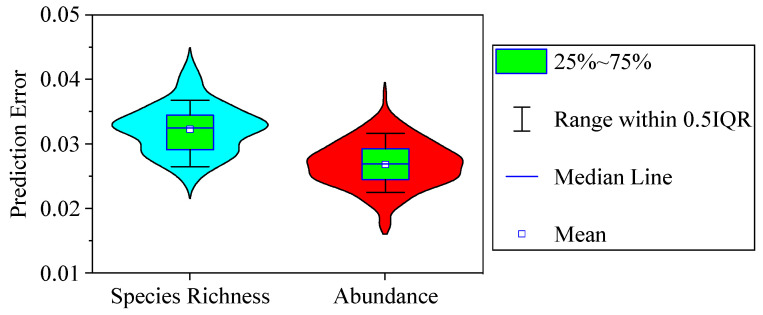
Violin plots showing the average errors between empirical and predicted phytoplankton species richness and abundance. The predicted values were calculated based on the CART, and the average predicted error was calculated with Equation (5).

The results showed that the average predicted error for phytoplankton species richness and abundance was 3.23% ± 0.38% (mean ± standard deviation) and 2.69% ± 0.35%, respectively (Figure 8). Therefore, CART can be used as a powerful method to analyze the response of phytoplankton to environmental variables and predict their growth in a changing environment.

## 4. Discussion

Based on a two-year investigation in a shallow temperate mountain river, we showed the temporal–spatial patterns of the phytoplankton community and built a regression tree to explain and predict the species diversity and abundance of phytoplankton in the river.

In the regression tree, we found DO concentration had a negative influence on phytoplankton species richness. Generally, DO concentration was positively related to water flow, especially in this shallow mountain river [40]. A fast-flowing river promoted the DO concentration but limited the growth of phytoplankton due to a poor habitat for growth, leading to a negative relationship between DO and species richness in this mountain river. DO concentrations in April and June 2020 in the present research were much lower than other months, since the underground spring might be the main water source, and there existed constructions along the river. Thus, the complex influence of DO on phytoplankton should be further explored. The values of pH also had negative influence on phytoplankton diversity in the regression tree. Ecologists have found that phytoplankton growth rate has a unimodal relationship with water pH and reaches the maximum value at about 8.3 to 8.5 [18,19]. In our study, the river was in a weak alkaline state since average pH values varied from 8.38 ± 0.23 to 9.03 ± 0.51, which were higher than the peak value of the previous studies [18,19]. Therefore, a weak alkaline state could promote the growth of phytoplankton, while high values of pH had a negative influence on the growth and diversity of phytoplankton.

We also observed that TN had a negative influence on phytoplankton species richness in the regression tree. The species number of phytoplankton generally increases with nutrient concentrations in oligotrophic systems, while it decreases with nutrient concentrations in eutrophic environments due to the “paradox of enrichment” [20,21,22]. In this research, TN concentration was high since the river originates from a few mountains covered by forest. In the regression tree, the first TN split was 1.423 mg/L, and it had positive influence on phytoplankton diversity, while the effects of the second TN split (4.01 mg/L) were negative. Therefore, TN had a unimodal relationship with phytoplankton species diversity. Our conclusions were consistent with previous studies [20,21,22].

In the final regression tree of phytoplankton abundance, TN, WT, and TP all had a positive influence during the first two TN splits, two TP splits, and one WT split. Ecologists have found that temperature variability interacts with nutrient supply to affect phytoplankton physiology and stoichiometry at the community level [12,41]. In most temperate regions, the temperature cycle was the main factor determining the seasonal fluctuations of phytoplankton community structure [42,43]. The mountain river in this study is a typical temperate river with seasonal temperature fluctuations (Table 1). Furthermore, the nutrient concentrations varied very substantially during the study. CART showed that TN, WT, and TP explained 39.40%, 15.70%, and 14.09%, respectively, of the variance in phytoplankton abundance. Therefore, nutrient and temperature were still the main variables affecting phytoplankton abundance in a fluctuating environment.

The signs of the effects of most environmental variables were changed during the CART, indicating complex relationships between the environment and phytoplankton in natural ecosystems. In the regression tree of phytoplankton diversity, three splits of TN were positive while three were negative. Both DO and ORP had two positive and two negative splits. TP had four positive and one negative split. WT had one positive and two negative splits, while Tur had one positive and one negative split. In the regression tree of phytoplankton abundance, TP had three positive and four negative splits. TN had four positive and two negative splits. WT had five positive and three negative splits, while DO had one positive and two negative splits. The changing signs of these effects during the CART indicate that some variables may promote phytoplankton diversity or abundance under certain conditions, while their influence may reverse under other combinations of environmental variable values.

All the environmental variables in CART explained 89.13% and 85.14% of the variances in total phytoplankton diversity and abundance, respectively, indicating a high influence of the environment on phytoplankton. CART could deal with a nonlinear relationship between environmental variables and plankton [35,36]. In addition, due to the fierce fluctuations of the environment in this mountain river, the combination of environmental variable values was temporally and spatially changed. CART accurately calculated the combination of these environmental variable values, as the average errors between the empirical and predicted values were 3.23% ± 0.38% for phytoplankton species diversity and 2.69% ± 0.35% for abundance. The prediction errors were also within 10% without logarithmic transformation. Therefore, CART provided a powerful way to predict the plankton community under a fluctuating environment. Bourel and Segura [44] showed that real phytoplankton data were accurately classified by CART with an error rate of about 0.05. Crisci et al. [45] also predicted phytoplankton biomass in periods of low turbidity with a classification error of 0.34, and the variables explained 46% of phytoplankton biomass variance. All these studies showed that machine learning is a superior method in analyzing the relationship between the environment and biology.

## 5. Conclusions

In the present study, we showed the temporal–spatial variations of phytoplankton species diversity and abundance based on a two-year field investigation in a shallow temperate mountain river where the environmental variables varied substantially. The quantitative contributions of each environmental variable on the phytoplankton community were explored using CART analysis. As a result, the following can be concluded:

(1) Both phytoplankton species diversity and abundance varied very substantially in this mountain river due to the fluctuation of the environment. Bacillariophyta, Cyanobacteria, and Chlorophyta were the dominant phyla, and *Microcystis aeruginosa* was the dominant species. Phytoplankton species diversity was higher while abundance was lower in 2020 than in 2019.
(2) CART analysis indicated that DO, ORP, TN, TP, and WT were the main variables that influenced phytoplankton species diversity, and they explained 36.00%, 13.81%, 11.35%, 9.96%, and 8.80%, respectively, of phytoplankton diversity variance.
(3) Phytoplankton abundance was mainly affected by TN, WT, and TP. Their proportional contributions to the overall explained variance in phytoplankton abundance were 39.40%, 15.70%, and 14.09%, respectively.
(4) The average errors between the empirical and predicted values were 3.23% ± 0.38% (mean ± standard error) for phytoplankton species diversity and 2.69% ± 0.35% for abundance. The phytoplankton community could be predicted precisely by CART analysis.
(5) Most environmental factors had a complex influence on phytoplankton diversity and abundance. Their effects were positive under some conditions but negative under other combinations of concentrations. Therefore, more machine learning methods should be used to explore their complex relationships.

The results and methodology in this study are of great significance in quantitatively understanding and exploring aquatic ecosystems.

## Figures and Tables

**Table 1:** Values of the main environmental variables at different months in the river; values are expressed as the mean ± standard deviation (minimum, maximum). WT: water temperature, DO: dissolved oxygen, EC: electrical conductivity, ORP: oxidation-reduction potential, Tur: turbidity, TN: total nitrogen, TP: total phosphorus.

Variables	WT	DO (mg/L)	EC (μS/cm)	pH	ORP	Tur (NTU)	TN (mg/L)	TP (mg/L)
April 2019	8.82 ± 4.15 (0.2, 13.5)	9.97 ± 1.05 (8.17, 12.92)	228.9 ± 57.9 (102.1, 326.6)	8.40 ± 0.21 (8.20, 8.88)	150.6 ± 32.0 (78.5, 183.2)	310.9 ± 215.0 (3.54, 602.9)	2.04 ± 1.92 (0.01, 5.20)	2.83 ± 2.31 (0.08, 9.70)
June 2019	15.34 ± 2.91 (10.5, 19.9)	10.42 ± 2.29 (6.62, 14.80)	265.9 ± 75.1 (166.9, 380.3)	8.38 ± 0.23 (7.95, 8.76)	187.96 ± 21.3 (135.7, 214.3)	102.4 ± 93.8 (1.08, 304.3)	7.65 ± 2.56 (0.62, 10.59)	0.35 ± 0.31 (0.02, 2.14)
August 2019	11.62 ± 2.23 (6.6, 14.7)	5.46 ± 2.24 (2.71, 9.81)	288.4 ± 74.8 (154.2, 371.6)	8.67 ± 0.54 (7.90, 10.81)	111.9 ± 28.4 (63.6, 173.5)	292.9 ± 251.8 (2.24, 600.0)	10.0 ± 1.12 (6.26, 11.38)	0.14 ± 0.13 (0.01, 0.47)
October 2019	5.19 ± 2.28 (0.6, 9.9)	3.62 ± 1.78 (0.02, 7.38)	255.2 ± 61.0 (142.8, 329.9)	8.63 ± 0.39 (8.27, 9.65)	139.2 ± 23.18 (67.9, 181.6)	352.8 ± 258.0 (7.93, 600.0)	2.18 ± 0.63 (1.03, 3.19)	0.57 ± 0.51 (0.07, 1.56)
April 2020	11.37 ± 3.63 (5.1, 17.4)	0.38 ± 0.30 (0.01, 1.65)	313.4 ± 131.4 (152.0, 782.0)	8.44 ± 2.07 (0.49, 11.56)	115.5 ± 35.1 (24.4, 182.9)	181.0 ± 156.6 (3.41, 452.9)	4.45 ± 1.54 (0.30, 6.41)	3.04 ± 0.30 (2.77, 3.70)
June 2020	14.02 ± 3.53 (7.0, 20.3)	0.61 ± 0.49 (0.01, 1.31)	320.5 ± 94.9 (147.9, 433.8)	8.41 ± 1.24 (3.69, 9.44)	117.2 ± 31.2 (71.7, 199.3)	88.7 ± 113.7 (1.56, 427.8)	4.28 ± 2.13 (0.01, 8.25)	0.19 ± 0.12 (0.06, 0.49)
August 2020	15.40 ± 2.66 (10.4, 19.8)	7.99 ± 0.44 (7.03, 8.97)	292.0 ± 89.7 (137.1, 417.4)	8.59 ± 0.31 (8.11, 9.46)	47.65 ± 18.58 (4.7, 72.9)	370.4 ± 266.2 (8.17, 629.0)	3.96 ± 1.77 (0.98, 7.62)	0.92 ± 0.65 (0.11, 1.97)
October 2020	6.71 ± 2.99 (0.0, 11.1)	10.94 ± 3.00 (8.80, 22.71)	282.8 ± 67.1 (151.9, 346.0)	9.03 ± 0.51 (8.35, 10.03)	82.99 ± 33.64 (7.3, 141.8)	141.5 ± 169.1 (0.90, 600.0)	3.22 ± 1.60 (0.54, 5.50)	0.40 ± 0.35 (0.01, 2.24)

## Data Availability

The data that support the findings of this study are available from the corresponding author upon reasonable request.
